# Embelin as a Novel Inhibitor of PKC in the Prevention of Platelet Activation and Thrombus Formation

**DOI:** 10.3390/jcm8101724

**Published:** 2019-10-18

**Authors:** Jiun Yi Li, Ray Jade Chen, Li Ting Huang, Tzu Yin Lee, Wan Jung Lu, Kuan Hung Lin

**Affiliations:** 1 Department of Medicine, Mackay Medical College, New Taipei City 252, Taiwan; jyl5891@gmail.com; 2 Department of Surgery, MacKay Memorial Hospital, Taipei 104, Taiwan; 3 Department of Pharmacology, School of Medicine, College of Medicine, Taipei Medical University, Taipei 110, Taiwan; tiffany4441@gmail.com (L.T.H.); d119103001@tmu.edu.tw (T.Y.L.); 4 Division of General Surgery, Department of Surgery, Taipei Medical University Hospital, Taipei 110, Taiwan; rayjchen@tmu.edu.tw; 5 Department of Surgery, School of Medicine, College of Medicine, Taipei Medical University, Taipei 110, Taiwan; 6 Department of Medical Research, Taipei Medical University Hospital, Taipei 110, Taiwan; 7 Graduate Institute of Metabolism and Obesity Sciences, College of Nutrition, Taipei Medical University, Taipei 110, Taiwan; 8 Institute of Biomedical Sciences, Mackay Medical College, New Taipei City 252, Taiwan

**Keywords:** embelin, granule release, platelet aggregation, PKC, thrombus formation

## Abstract

Embelin is a quinone derivative and found in the fruits of *Embelia ribes* Burm.f. Embelin has been identified as a small molecular inhibitor of X-chromosome-linked inhibitor of apoptosis proteins, and has multiple biological activities, including antioxidation, anti-inflammation, and antitumor effects. However, the effect of embelin in platelets remains unclear. Thus, this study investigated the antiplatelet mechanism of embelin. Our data revealed that embelin could inhibit platelet aggregation induced by various agonists, including the protein kinase C (PKC) activator phorbol 12,13-dibutyrate (PDBu). Embelin, as well as the PKC inhibitor Ro 31-8220, markedly reduced PDBu-mediated phosphorylation of the PKC substrate, suggesting that embelin may be a PKC inhibitor for platelets. Embelin could block PKC downstream signaling and events, including the inhibition of protein kinase B and mitogen-activated protein kinase activation, granule release, and glycoprotein IIbIIIa activation. Moreover, embelin could delay thrombus formation in the mesenteric microvessels of mice, but did not significantly affect the tail bleeding time. In conclusion, we demonstrated that embelin is a PKC inhibitor and possesses antiplatelet and antithrombotic effects. The further analysis is necessary to more accurately determine clinical therapeutic potential of embelin in all clinical thromboembolic events with disturbance of thrombocyte function.

## 1. Introduction

Platelets and blood coagulation are involved in hemostasis. When a blood vessel is injured, rolling platelets adhere to the injured site of the vessel, and are activated by the exposed extracellular matrix such as collagen and vWF, which causes primary platelet activation by the binding to glycoprotein VI (GPVI) and GPIb-V-IX complex, respectively. Then, the activated platelets release ADP and thromboxane A_2_ that can amplify platelet activation by the autocrine or paracrine manner and further recruit circulating platelets to engage in the process of platelet plug formation at the injured site of the vessel. In addition, the extrinsic pathway of blood coagulation cascade can be initiated by the interaction of plasma VII with tissue factor (TF) exposed after vascular injury. The TF-VIIa complex can activate factors X and IX. Subsequently, the Xa generates small amounts of thrombin from prothrombin, and is rapidly inactivated by tissue factor pathway inhibitor. Although this amount of thrombin is not sufficient to initiate significant fibrin polymerization, it can activate platelets and the intrinsic pathway (factors V, VIII, and XI). Once platelets are activated, factors Va and VIIIa are rapidly localized on the platelet membrane surface. Subsequently, the factor IXa generated by the TF-VIIa complex or XIa can bind to VIIIa and form the VIIIa-IXa complex, which amplifies the generation of factor Xa on the platelet membrane surface. Once factor Xa gets associated with Va on the platelet surface to generate a burst of thrombin, it is followed by fibrin formation and factor XIII activation, finally leading to the formation of a firm platelet plug and subsequent hemostasis. Thus, Hemostasis requires platelets and the coagulation system [1,2]. Platelets are also involved in various pathological processes, such as inflammation, atherothrombosis, and tumor metastasis. Thus, the inhibition of platelet activation may impede or attenuate the progression of these diseases.

Embelin, a quinone derivative, is found in the fruits of *Embelia ribes* Burm.f. It is reported to have multiple biological activities. Embelin could protect nephrons against cisplatin-induced damage caused by oxidative stress and inflammation [3]. Embelin also protects against myocardial ischemia–reperfusion injury and transient global ischemia-induced brain damage [4,5]. In addition, embelin has an immunosuppressive property that attenuates autoimmune encephalomyelitis through the transforming growth factor-β/β-catenin and signal transducer and activator of transcription 3 (STAT3) signaling pathways [6]. Embelin protects pancreatic β-cells in streptozotocin-induced diabetes [7,8]. Moreover, embelin has been observed to suppress tumor growth through interleukin 6/STAT3 signaling in various cancer types [9,10,11], and can also inhibit tumor metastasis [12,13]. This evidence suggests that embelin possesses multiple biological activities.

However, the role of embelin on platelet activation and thrombus formation has not been investigated. Therefore, we then further investigated the mechanism underlying the embelin-mediated inhibition of platelet activation.

## 2. Materials and Methods

### 2.1. Materials

Embelin and convulxin were purchased from Cayman Chemical (Ann Arbor, MI, USA). Collagen, thrombin, and U46619 were purchased from Chrono-log (Havertown, PA, USA). Phorbol 12,13-dibutyrate (PDBu), luciferase, luciferin, and fluorescein sodium were purchased from Sigma (St. Louis, MO, USA). Phycoerythrin (PE)-conjugated anti-P-selectin and fluorescein isothiocyanate (FITC)-conjugated PAC-1 antibodies were purchased from BioLegend (San Diego, CA, USA). The anti-phospho-(serine (Ser)) protein kinase C (PKC) substrate, anti-phospho-p38 mitogen-activated protein kinases (MAPKs) (Ser^180^/tyrosine (Tyr)^182^), anti-phospho-p44/42 MAPKs (extracellular signal-regulated kinases (ERKs)1/2, threonine (Thr)^202^/Tyr^204^), anti-c-Jun N-terminal kinases (JNKs), anti-phospho-protein kinase B (Akt) (Ser^473^) polyclonal antibodies (pAbs), and anti-p38 MAPK, anti-p44/42 MAPK, anti-phospho JNKs (Thr^183^/Tyr^185^), and anti-Akt monoclonal antibodies were purchased from Cell Signaling Technology (Beverly, MA, USA). The antipleckstrin (p47) pAb was purchased from GeneTex (Irvine, CA, USA). The Hybond-P polyvinylidene difluoride (PVDF) membrane, an enhanced chemiluminescence (ECL), and the horseradish peroxidase (HRP)-conjugated donkey antirabbit and sheep antimouse immunoglobulin G were purchased from GE Healthcare Life Sciences (Buckinghamshire, UK). Embelin was dissolved in dimethyl sulfoxide (DMSO) and stored at 4 °C until use.

### 2.2. Preparation of Washed Human Platelets

This study was approved by the Taipei Medical University-Joint Institutional Review Board (TMU-JIRB No. N201810057, 23 November 2018) and conformed to the principles outlined in the Declaration of Helsinki. All volunteers provided informed consent. Human platelet suspensions were prepared as previously described [14,15,16,17], according to the guideline of International Society on Thrombosis and Haemostasis (ISTH). Before blood collection, the subjects, who had taken no medicine such as aspirin and other NSAIDs or thienopyridines during the preceding 2 weeks, needed to take a short rest period, and refrain from smoking and drinking coffee for at least 30 min and 2 h. With a butterfly, blood was drawn from healthy volunteers, and the first 1–2 mL of blood were discarded to decrease the contamination with tissue factors and trace amounts of thrombin. Blood samples were immediately collected in plastic tubes (polypropylene) and mixed with an acid-citrate-dextrose (ACD) solution (9:1, v/v). Blood samples were allowed to rest at room temperature for 15 min. Then, centrifugation of blood samples at 250 × *g* was conducted for 10 min. In this step, the centrifugal slow brake must be applied during rotor deceleration to prevent red blood cells rising from the bottom layer. The upper layer containing platelet-rich plasma (PRP) was carefully collected without disturbing the middle layer containing white blood cells. The PRP was supplemented with 0.5 μM prostaglandin E_1_ and 6.4 IU/mL heparin for 10 min at 37 °C. Then, the PRP was centrifuged at 2200 × *g* for 10 min. After centrifugation, the supernatant consisting of platelet-poor plasma (PPP) was discarded, and all traces of plasma from the tube walls or near the platelet pellet was carefully removed to avoid any generation of thrombin during the subsequent washing steps. The platelet pellet was gently resuspended in Tyrode’s solution containing 3.5 mg/mL bovine serum albumin (BSA), 0.5 μM prostaglandin E_1_ (PGE_1_), and 6.4 IU/mL heparin to obtain the first washed human platelet suspension. After 10 min incubation at 37 ℃, platelet suspension was immediately centrifuged at 1900 × *g* for 8 min. The platelet pellet was gently resuspended in Tyrode’s solution containing 3.5 mg/mL BSA and 0.5 μM PGE1. A few microliters of platelet suspension were removed and counted using an automatic blood cell counter. After 10 min incubation at 37 ℃, the second washed platelet suspension was immediately centrifuged at 1900 × *g* for 8 min. The platelet pellet was resuspended in Tyrode’s solution containing 3.5 mg/mL BSA and 0.02 U/mL apyrase, and platelet count was adjusted to 3.6 × 108 cells/mL. The final concentration of Ca^2+^ in platelet suspension was 1 mM. Before testing, these platelet suspensions were allowed to rest for at least 30 min at 37 ℃.

### 2.3. Platelet Aggregation

A turbidimetric method was applied to measure platelet aggregation by using a lumi-aggregometer (Payton, Scarborough, Ontario, Canada) [14]. Before testing, the light transmission of platelet suspension (3.6 × 108 cells/mL) and Tyrode’s solution was set as 0% and 100%, respectively. Platelet aggregation was performed at 37 ℃ with magnetic stirring (1000 rpm). Before treatment, baseline tracings for light transmission were observed for stability for at least 1 min. Then, platelet suspension was preincubated with embelin (50, 75, and 100 μM) or an isovolumetric solvent control (0.1% DMSO) for 3 min prior to agonist administration. The platelet aggregation was recorded for 6 min (collagen, convulxin, thrombin, and U46619) or 10 min (PDBu).

### 2.4. Immunoblotting Study

1.2 × 10^9^ platelets/mL were preincubated with embelin (75 and 100 μM), 2 μM Ro 31-8220, or 0.1% DMSO for 3 min prior to PDBu (150 nM) administration for 20 min to stimulate platelet activation. After the reaction, the platelets were immediately resuspended in 200 μL of lysis buffer for 1 h. Lysates were centrifuged at 5000 × *g* for 5 min. The extracted protein (80 μg) was separated using sodium dodecyl sulfate polyacrylamide gel electrophoresis on an 12% gel; the separated proteins were then transferred onto PVDF membrane through semidry transfer (Bio-Rad Laboratories, Hercules, CA, USA). Membranes were blocked with 5% BSA for 1 h, washed for 3 times with TBST (10 mM Tris base, 100 mM NaCl, and 0.01% Tween 20), and then stained with various primary antibodies specific to the target proteins for 1 h. Membranes were incubated with HRP-conjugated antimouse IgG or antirabbit IgG (diluted 1:3000 in TBST) for 1 h, and then developed using ECL kit. The intensity of the immunoreactive bands was quantified using a videodensitometer and the Bio-profil Biolight software, version V2000.01 (Vilber Lourmat, Marne-la-Vallée, France).

### 2.5. Adenosine Triphosphate Release Measured Using a Microplate Reader

Luciferase and luciferin were used to detect adenosine triphosphate (ATP) release. This method was described previously [18]. Briefly, 3.6 × 10^8^ platelets/mL were preincubated with luciferase and luciferin, and then with embelin (75 and 100 μM) or an isovolumetric solvent control (0.1% DMSO) for 3 min prior to PDBu administration. After mixing, the mixture was transferred to a white-walled 96-well plate. The reaction was allowed to proceed for 30 min at 37 °C and the intensity of luminescence was detected every minute using a Synergy H1 microplate reader (BioTek, Winooski, VT, USA).

### 2.6. Flow Cytometry

P-selectin secretion and GPIIbIIIa activation were determined by flow cytometry as described previously [18]. Briefly, 3.6 × 10^8^ platelets/mL were preincubated with embelin (75 and 100 μM) or 0.1% DMSO for 3 min; subsequently, PDBu was added for 20 min in glass cuvettes at 37 °C. After the reactions, the samples were fixed with 1% paraformaldehyde for 1 h at 4 °C, washed, and labeled with a PE–P-selectin or FITC–PAC-1 antibody for 30 min. After centrifugation and washing, all of the samples were resuspended with 1 mL of phosphate-buffered saline and were then immediately analyzed on a FACSCanto II Flow cytometer (BD Biosciences, Franklin Lakes, NJ, USA). The platelets were identified and gated by their characteristic forward and side scatter properties, and 10,000 platelets were analyzed from each sample. All experiments were performed at least three times to ensure reliability.

### 2.7. Determination of Lactate Dehydrogenase

A CytoTox 96 nonradioactive cytotoxicity assay kit from Promega (Madison, WI, USA) was used to measure lactate dehydrogenase (LDH) release. In total, 3.6 × 10^8^ platelets/mL were preincubated with embelin (75 and 100 μM) or a solvent control (0.1% DMSO) for 20 min at 37 °C. After centrifugation was conducted, the supernatant was collected to measure the LDH level according to the manufacturer’s protocol (Promega). LDH activity was expressed as the percentage of total enzyme activity, which was obtained when platelets were lysed with Triton X-100 (0.5%). The LDH level was measured at a wavelength of 490 nm by using a Synergy H1 microplate reader (BioTek, Winooski, VT, USA).

### 2.8. Animals

ICR mice (aged 5–6 weeks, male, weighing 20–25 g) were obtained from BioLasco (Taipei, Taiwan). All procedures were approved by the Affidavit of Approval of Animal Use Protocol of Shin Kong Wu Ho-Su Memorial Hospital (Approval No. SKH107001, 21 September 2017) and were in accordance with the Guide for the Care and Use of Laboratory Animals (Eighth Edition, 2011).

### 2.9. Fluorescein-Sodium-Induced Platelet Thrombus Formation in The Mesenteric Microvessels of Mice

Thrombus formation was assessed as previously described [19]. Briefly, mice were anesthetized using a mixture containing 75% air and 3% isoflurane maintained in 25% oxygen at a flow rate of 1.5–2 L/min; the mouse external jugular vein was cannulated with a polyethylene-10 tube for administration of dye and drugs intravenously. A segment of the small intestine was placed onto a transparent culture dish for microscopic observation. The venules (30–40 mm) were selected for irradiation to produce a microthrombus. Filtered light for which wavelengths <520 nm had been eliminated was used to irradiate a microvessel. Embelin (11.5 and 23 mg/kg) or aspirin (20 mg/kg) was administered 1 min after sodium fluorescein (15 mg/kg) addition, and the time required to occlude the microvessel (occlusion time) was recorded. The dose for mice was accordingly converted from the dose for humans [20].

### 2.10. Tail Bleeding Time

Mice were anesthetized using a mixture containing 75% air and 3% isoflurane maintained in 25% oxygen at a flow rate of 1.5–2 L/min. Then, DMSO (solvent control), embelin (11.5 and 23 mg/kg), or aspirin (20 mg/kg) were intraperitoneally administrated for 30 min. A distal 3-mm segment of the tail was amputated with a scalpel. The bleeding tail stump was immersed in saline and bleeding was immediately monitored for 10 min. The bleeding time was obtained until no sign of bleeding was observed for at least 10 s. The dose for mice was accordingly converted from the dose for humans [20].

### 2.11. Data Analysis

Results are expressed as means ± standard error of the mean (SEM) and are accompanied by the number of observations (*n*). The values of *n* refer to the number of experiments in which blood samples were collected from different donors. Results were analyzed using analysis of variance (ANOVA). When ANOVA indicated significant differences among groups, the groups were then compared using the Newman–Keuls method. Values of *P* < 0.05 were considered statistically significant.

## 3. Results

### 3.1. Embelin Inhibited Human Platelet Aggregation

**Figure 1:**
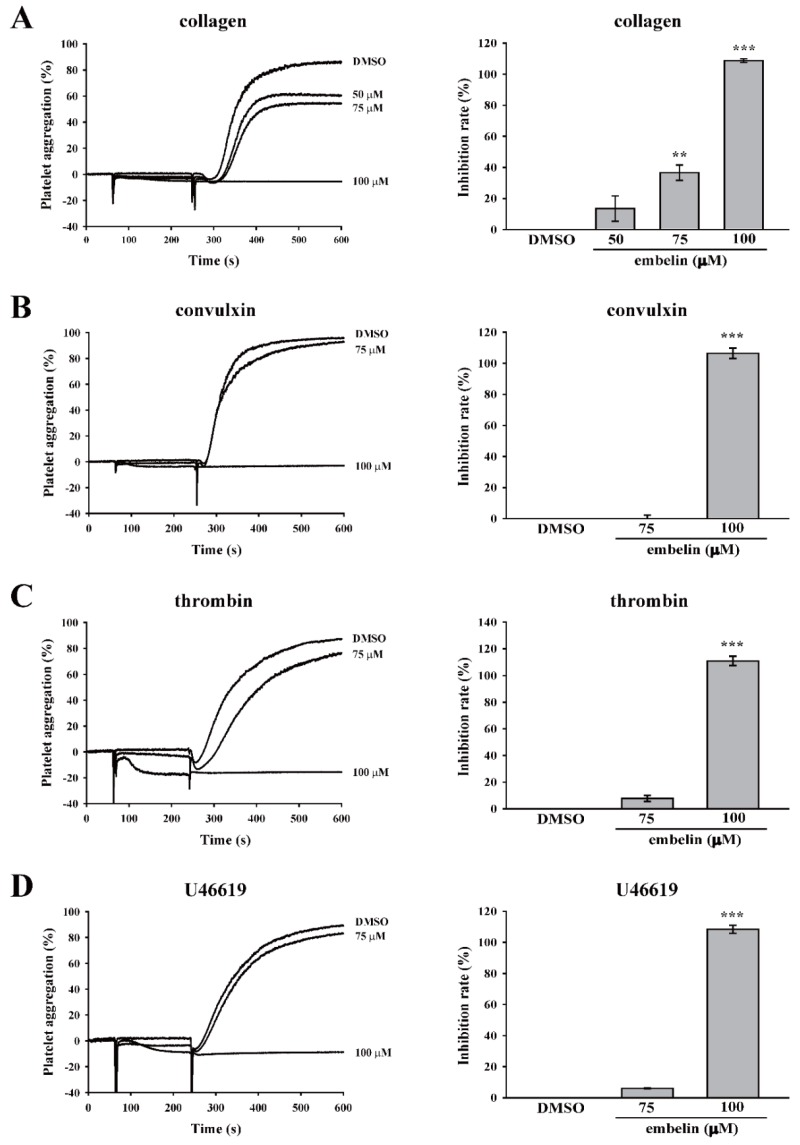
Embelin reduced various agonist-induced human platelet aggregations. Human washed platelets (3.6 × 10^8^ cells/mL) were pretreated with dimethyl sulfoxide (DMSO) (solvent control) or embelin (50–100 μM), followed by stimulation with (**A**) collagen (1 μg/mL), (**B**) convulxin (10 ng/mL), (**C**) thrombin (0.02 U/mL), and (**D**) U46619 (1 μM) to induce platelet aggregation. **Left panels** indicate the tracing curve of platelet aggregation, and **right panels** indicate the statistical bar graphs of inhibition rate (%). Data (**A–D**) are presented as means ± standard error of the mean (SEM) (*n* = 3). **P < 0.01 and ***P < 0.001, compared with the DMSO (solvent control) group.

In this study, various platelet agonists, including collagen, convulxin, thrombin, and U46619, were used to determine the effect of embelin on platelet aggregation. The data showed that there is no difference between Tyrode’s solution (control) and dimethyl sulfoxide (DMSO, solvent control) on platelet aggregation induced by these four platelet agonists (Supplementary Figure S1). As shown in Figure 1A–D, the data revealed that embelin at 75 μM displayed only a partial inhibition of platelet aggregation induced by collagen and no significant inhibition of platelet aggregation induced by convulxin, thrombin, and U446619, but embelin at 100 μM almost completely inhibited platelet aggregation induced by these four agonists. These results suggested that embelin may not directly block the agonist receptors on the surface of platelets. Thus, we supposed that embelin might act on the common pathways of platelet activation, such as the PKC pathway. Thus, we further determined the effect of embelin on the PKC pathway, the common platelet activation pathway, in subsequent experiments. In addition, the result of LDH assay revealed that 75 and 100 μM embelin did not exhibit cytotoxicity in human platelets.

### 3.2. Embelin Attenuated Platelet Activation Through the Direct Inhibition of PKC

**Figure 2:**
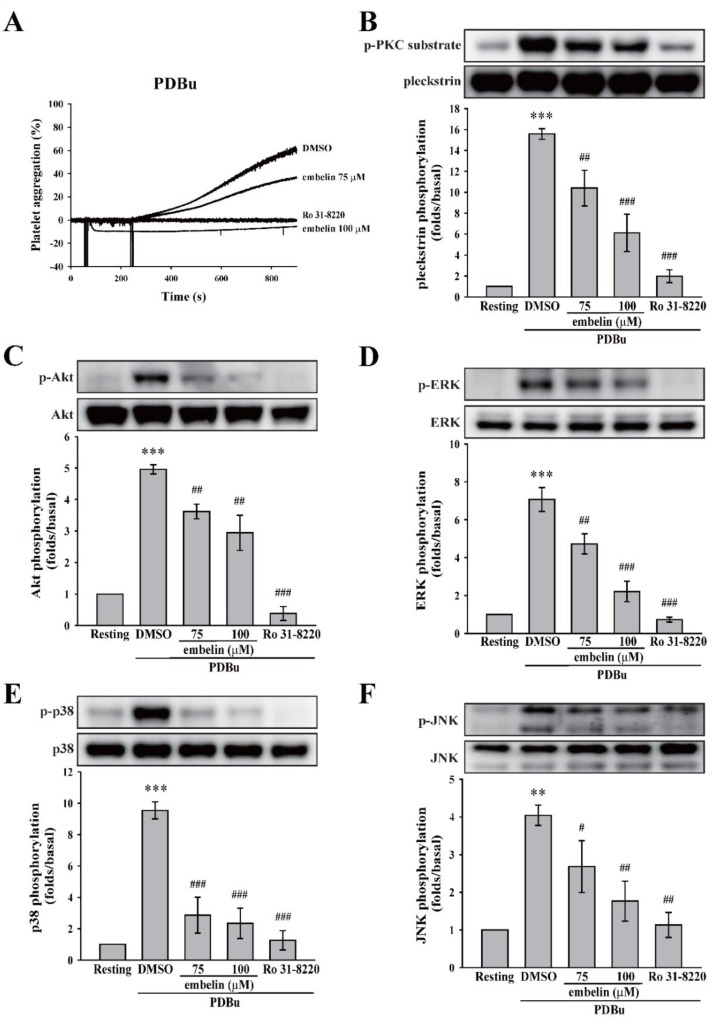
Embelin inhibited protein kinase C (PKC) activity and its downstream signaling. (**A**) Human washed platelets (3.6 × 10^8^ cells/mL) were pretreated with DMSO (solvent control), embelin (75 and 100 μM), or the PKC inhibitor Ro 31-8220 (2 μM), followed by the stimulation of phorbol 12,13-dibutyrate (PDBu) (150 nM) to induce platelet aggregation. (**B–F**) After the reaction, platelet lysates were collected and then subjected to Western blotting. Specific antibodies were used to detect PKC, protein kinase B (Akt), the extracellular signal-regulated kinase (ERK), p38 mitogen-activated protein kinase (p38), and c-Jun N-terminal kinase (JNK). Profiles (**A**) are representative examples of three similar experiments. Data (**B–F**) are presented as means ± SEM (*n* = 4). **P < 0.01 and ***P < 0.001, compared with the resting group. #*P* < 0.05, ##*P* < 0.01, and ###*P* < 0.001, compared with the PDBu-treated (positive control) group.

As previously mentioned, PKC pathway is commonly involved in platelet activation. Thus, we first determined whether embelin can affect the PKC activator PDBu-induced platelet aggregation. Likely, no difference between Tyrode’s solution (control) and dimethyl sulfoxide (DMSO, solvent control) on platelet aggregation induced by PDBu was observed (supplemental Figure S2A). As demonstrated in Figure 2A and supplemental Figure S2B, embelin (75 and 100 μM), as well as the PKC inhibitor Ro 31-8220, significantly inhibited PDBu-induced platelet aggregation, indicating that it could inhibit PKC or its downstream signaling. To confirm whether embelin could block PKC activation, the phosphorylation of p47 protein (pleckstrin), a major PKC substrate (approximately 47 kD) that is widely used to determine PKC activity, was detected through Western blotting. Figure 2B reveals that embelin (75 and 100 μM), as well as the PKC inhibitor Ro 31-8220, significantly prevented PDBu-induced p47 phosphorylation, suggesting that embelin directly inhibited PKC activity. In addition, PKC downstream signaling, including Akt and MAPKs (ERK, p38 MAPK, and JNK) that are responsible for granule release, thromboxane A_2_ formation, and glycoprotein (GP) IIbIIIa [21,22] were also determined. As shown in Figure 2C–F, embelin (75 and 100 μM) and Ro 31-8220 reduced the phosphorylation of Akt and MAPKs. These findings suggest that embelin attenuated platelet activation through the direct inhibition of PKC activity.

### 3.3. Embelin Reduced PKC-Mediated Granule Release and GPIIbIIIa Activation

**Figure 3:**
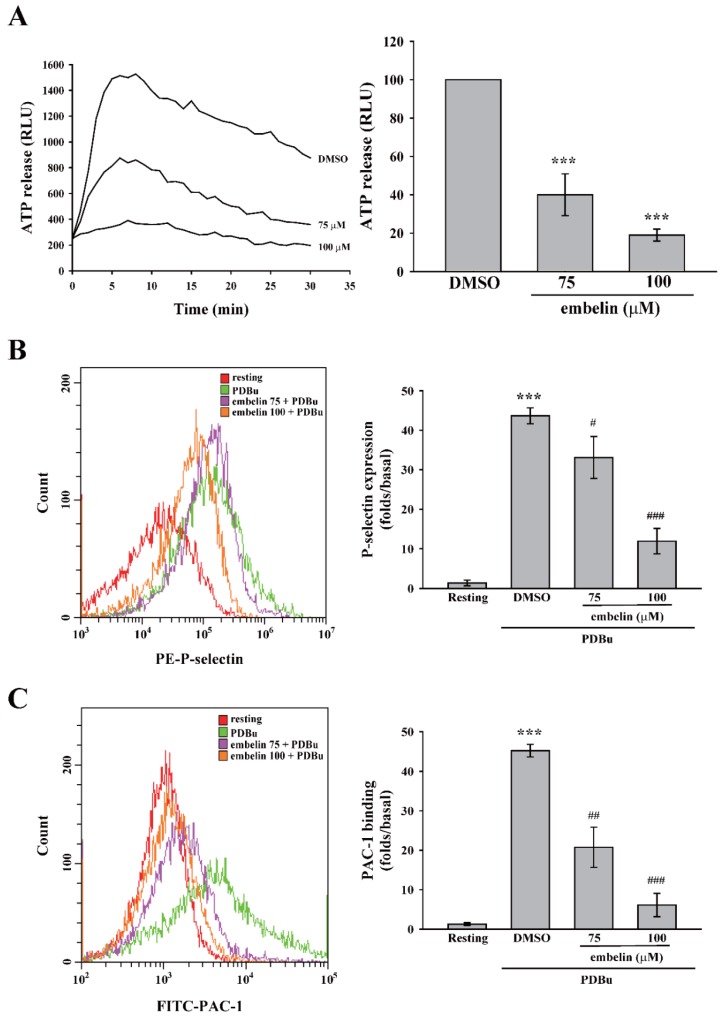
Embelin attenuated granule release and glycoprotein IIbIIIa (GPIIbIIIa) activation. Human washed platelets (3.6 × 10^8^ cells/mL) were pretreated with DMSO (solvent control) or embelin (75 and 100 μM), followed by stimulation with PDBu (150 nM) to induce platelet activation. (**A**) Luciferase and luciferin were added to detect adenosine triphosphate (ATP) release using a microplate reader. (**B**,**C**) Phycoerythrin (PE)-conjugated with P-selectin and fluorescein isothiocyanate (FITC)-conjugated PAC-1 antibodies were used to detect P-selectin secretion and GPIIbIIIa activation, respectively, using flow cytometry. Data (**A**) are presented as means ± SEM (*n* = 3). ***P < 0.001, compared with the DMSO (solvent control) group. Data (**B**,**C**) are presented as means ± SEM (*n* = 3). ***P < 0.001, compared with the resting group. #*P* < 0.05, ##*P* < 0.01, and ###*P* < 0.001, compared with the PDBu-treated (positive control) group.

PKC is involved in platelet activation, including granule release and GPIIbIIIa activation [21,22]. Thus, we investigated the role of embelin in these events. Figure 3A,B illustrates that embelin (75 and 100 μM) significantly reduced PDBu-induced ATP release and P-selectin secretion, suggesting that embelin inhibited PKC-dependent platelet granule release. In addition, embelin (75 and 100 μM) inhibited PDBu-induced GPIIbIIIa activation (Figure 3C). This evidence supports the findings that, with its inhibitory effect on PKC activity, embelin can prevent PKC-associated platelet activation, including granule release and GPIIbIIIa activation.

### 3.4. Embelin Exerted Antithrombotic Activity Without Bleeding Side Effects

**Figure 4:**
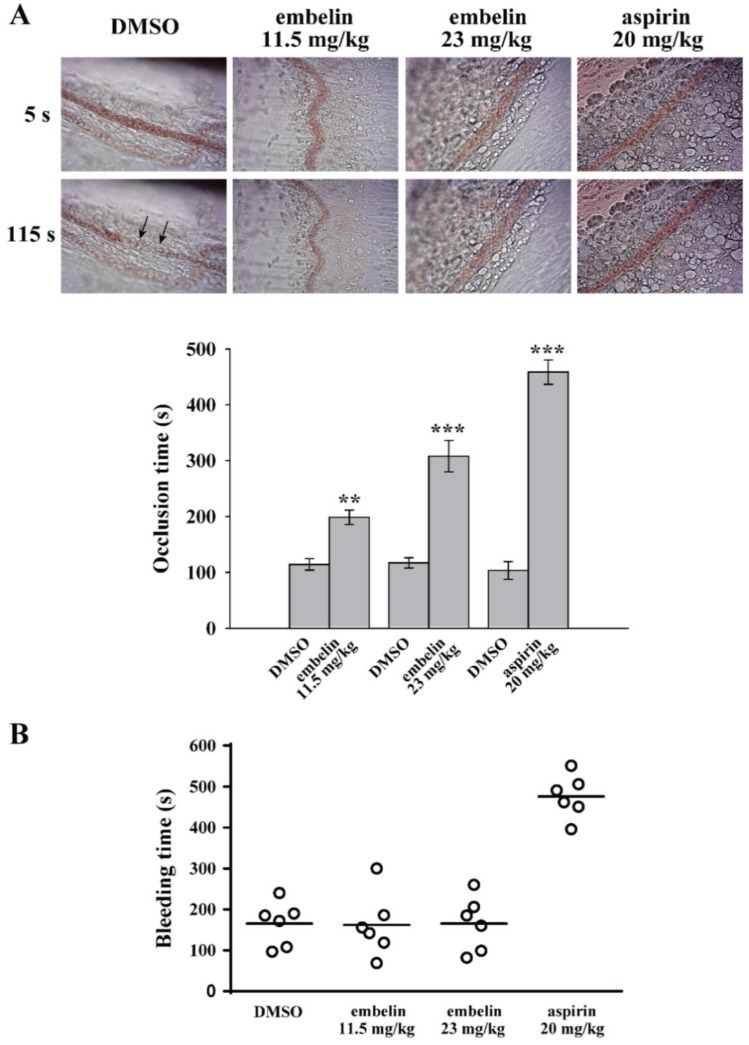
Embelin exerted antithrombotic effects without affecting hemostasis. (**A**) Mice received an intravenous bolus of DMSO, embelin (11.5 and 23 mg/kg), and aspirin (20 mg/kg) for 30 min, followed by an injection of fluorescein sodium. The mesenteric venules were then irradiated to induce vessel damage and microthrombus formation. The arrows indicate occlusion of the mesenteric venule (*n* = 6). (**B**) The bleeding was continually monitored through the transection of mice tails after 30 min of intraperitoneal administration of either DMSO, embelin (11.5 and 23 mg/kg), and aspirin (20 mg/kg). Subsequently, the bleeding time was recorded until no sign of bleeding was observed for at least 10 s. Each point in the scatter plots graph represents a mouse (*n* = 6). Data (A) are presented as mean ± SEM (*n* = 6). **P < 0.01 and ***P < 0.001 compared with the DMSO group.

In addition to the in vitro antiplatelet effect, the in vivo antithrombotic effect of embelin was determined. In the thrombotic animal model, mice were intravenously administered fluorescein sodium and the mesenteric microvessels of the mice were subsequently exposed to ultraviolet irradiation, which damaged the endothelium and caused platelet thrombus formation and vascular occlusion. The vascular occlusion was continually monitored using a microscope with a camera. The vessels’ occlusion times were recorded. Figure 4A (top panel) reveals that vessel occlusion (arrows) occurred for approximately 115 s in the DMSO group. Embelin (11.5 and 23 mg/kg) and 20 mg/kg aspirin significantly delayed the occlusion time by approximately 84.5 ± 2.8 s (*P* < 0.01, *n* = 6), 191.0 ± 18.9 s (*P* < 0.001, *n* = 6), and 355.3 ± 5.9 s (*P* < 0.001, *n* = 6), respectively (Figure 4A, bottom panel). In addition, embelin’s effect on hemostasis was determined by the tail bleeding time. The data demonstrated that the group administered embelin (11.5 and 23 mg/kg) did not significantly prolong the bleeding time, compared with the DMSO group (165.3 ± 22.0 s, *n* = 6) (Figure 4B). Unlike embelin, aspirin (20 mg/kg)-treated group markedly prolonged the bleeding time (476.2 ± 21.6 s; *P* < 0.001, *n* = 6). These findings suggest that embelin exerted a safe and potent antithrombotic effect without affecting hemostasis.

## 4. Discussion

**Figure 5:**
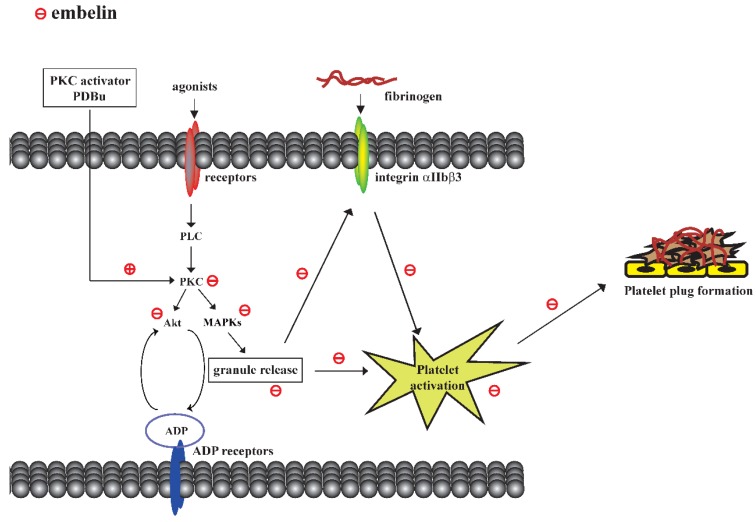
Scheme of the mechanism underlying embelin-mediated inhibition of platelet activation and thrombus formation.

This study demonstrated that embelin possesses antiplatelet and antithrombotic effects through direct inhibition of PKC activity followed by the blockade of granule release and GPIIbIIIa activation. Embelin thus reduced platelet activation and thrombus formation (Figure 5).

Embelin was previously identified as a small molecular inhibitor of the X-linked inhibitor of apoptosis proteins that can cause cell death through activating caspase-9 and subsequent cell apoptosis in prostate cancer cells with high levels of XIAP [23]. In human platelets, we found that embelin could inhibit platelet aggregation induced by various agonists including collagen, convulxin, thrombin, and U46619. These results suggested that embelin may not directly block the agonist receptors on the surface of platelets. Moreover, LDH assay has excluded the possibility that embelin may directly lead to platelet damage, which may cause themselves to be unresponsive to platelet agonists. Thus, we supposed that embelin might act on the common pathways of platelet activation, such as the PKC pathway. Indeed, our results revealed that embelin was a PKC inhibitor that could effectively block platelet aggregation and thrombus formation. PKC plays an essential role in platelet activation and thrombus formation [21,24]. PKC reportedly regulates platelet granule release and GPIIbIIIa activation [21,22]. We found that PKC inhibition by embelin could effectively block ATP release, P-selectin secretion, and GPIIbIIIa activation of human platelets in vitro, and prevent thrombus formation in vivo.

In addition, our data revealed that the PKC activator PDBu could markedly induce the activation of Akt and MAPKs, including ERK, p38 MAPK, and JNK, which was inhibited by embelin. Akt signaling has been reported to be involved in fibrinogen binding and platelet aggregation [25,26]. Moreover, the adenosine diphosphate (ADP)-stimulated G_i_ signaling pathway played an essential role in Akt activation in one study [27]. This evidence indicates that embelin may have reduced dense granule release, thereby blocking Akt activation and platelet aggregation; ERK2, p38 MAPK, and JNK1 were also involved in platelet adhesion, granule release, aggregation, and thrombus formation [28]. Furthermore, PKC signaling was reported to be essential for the secretion of thromboxane A_2_ via ERK and p38 MAPK [21]. The interaction of JNK and PKC in platelets remains unclear, though PKC has been reported to activate JNK in other cells [29,30]. Adam et al. reported that JNK1^−/−^ platelets partially impaired PKC activity [28]. However, the possibility that JNK1 acts downstream from PKCs in platelets cannot be excluded because JNK1^−/−^ platelets were observed to not affect the phosphorylation of PKC substrates induced by phorbol-12-myristate 13-acetate (a PKC activator) [28]. In the present study, we discerned that either the PKC inhibitor Ro 31-8220 or embelin could block the PKC activator PDBu-mediated phosphorylation of the PKC substrate, and reduced JNK phosphorylation. These observations suggested that the interaction of PKC and JNK was mutual. Taken together, the evidence suggests that PKC inhibition by embelin could prevent the activation of MAPKs and was followed by a blockade of granule release and thromboxane A_2_ secretion, finally leading to the inhibition of platelet activation and thrombus formation. On the other hand, embelin reportedly has the anti-inflammatory and antitumor effects through inhibition of IL6/STAT3 and NF-κB signaling pathways [6,9,10,11,31]. Previous studies have reported that STAT3 and NF-κB are involved in platelet activation through non-transcriptional activity [32,33] and that inhibition of STAT3 and NF-κB could impair platelet activation [32,33,34,35]. IL6 was also reported to induce platelet activation [36]; in addition, embelin could reduce lipid peroxidation and scavenge free radicals [37,38]. Studies have showed that reactive oxygen species play a critical role in platelet activation [39,40]. Taken together, these effects of embelin might support its antiplatelet effect, but further investigation are needed to confirm these effects of embelin on platelet function.

Atherosclerosis, a major cause of cardiovascular diseases such as stroke and heart attack [41,42,43], is a complicated inflammatory process involving lipid accumulation and the activities of various cells, including macrophages, leucocytes, endothelial cells, smooth muscle cells, and platelets [41,42,43]. Hypercholesterolemia cause lipid or LDL accumulation in the arterial intima, where LDL undergoes oxidative modification, which induces inflammatory responses characterized by chemokine secretion and altered expression of adhesion molecules, such as vascular cell adhesion molecule-1, on focal endothelial cells [44,45]. These inflammatory responses further recruit circulating monocytes in the intima where they differentiate into macrophages, and form foam cells when macrophage intake naïve or modified lipid. Foam cells can amplify lipid accumulation and modification [44,45]. Moreover, activated macrophage further stimulate immune system and amplify the inflammation process that enhances the progression of atherosclerosis [44,45]. In addition, platelets are also involved in the initiation of atherosclerosis by interacting with activated endothelial cells and recruiting monocytes. Moreover, it was generally accepted that rupture or erosion of advanced atherosclerotic lesions initiates platelet activation and aggregation on the thrombogenic surface of a disrupted atherosclerotic plaque [46]. A previous study also demonstrated that platelet scavenger receptor class B type 1 increased platelet hyperreactivity and accelerated thrombosis under hyperlipidemia due to increased platelet cholesterol content [47]. Platelet P-selectin was reported to be essential during the interaction of activated platelets with atherosclerotic arteries [48]. In addition, platelet adhesion through GPIb and GPIIbIIIa was also reported to be critical for the initiation of atherosclerotic lesion formation [49]. In addition, activated platelets can release and deposit chemokines on vascular cell surfaces, which trigger atherogenic recruitment of vascular cells. For example, activated platelets secreted interleukin-1β (IL-1 β) and expressed CD40 ligand that can further induce endothelial activation and secretion of chemokines, such as monocyte chemoattractant protein-1 and IL-8, thereby recruiting leucocytes and producing inflammatory responses [50,51]. Moreover, neutralization or genetic deletion of CD40 ligand has shown a reduction of the progression of atherosclerotic lesion [52,53]. These lines of evidence revealed that the inhibition of platelet activation exhibit a benefit in atherosclerosis. In the present study, our data revealed that embelin could reduce P-selectin secretion and GPIIbIIIa activation, at least in part, through PKC inhibition, eventually inhibiting platelet activation. Moreover, embelin was reported to reduce body weight gain, blood pressure, and serum lipid levels and increase superoxide dismutase, catalase, and glutathione levels in high-fat diet-induced obesity in rats [54]. It also reduced lipid peroxidation and scavenged free radicals [37,38]. These observations revealed that embelin may has a potential role in the prevention of atherosclerosis.

In addition, PKC isoforms were thought to be involved in regulation of monocyte–EC interaction [55]. PKCβ has been reported to promote vascular inflammation and exacerbate atherosclerosis in diabetic ApoE null mice [56]. Durpès et al. also reported that PKCβ activation cause endothelial dysfunction and diabetic atherosclerosis by inhibiting IL-18 binding protein [57]. However, we for the first time demonstrated that embelin is a PKC inhibitor in human platelets. Furthermore, a previous study reported that embelin has anti-diabetic activity via reducing intracellular pro-inflammatory mediators, lipid profile, and oxidative stress [8]. Whether embelin can inhibit interaction of monocytes and endothelial cells by inhibition of PKCβ and subsequently improve diabetic atherosclerosis needs to be further investigated. On the other hand, IL6/STAT3 and NF-κB signaling pathways have been reported to be important regulators in the progression of atherosclerosis, including endothelial dysfunction, cytokine secretion, macrophage polarization, foam cell formation, proliferation of vascular smooth muscle cells, and cell death [58,59,60,61,62]. Although, embelin has been reported to inhibit IL6/STAT3 and NF-κB signaling pathways [6,9,10,11,31], whether embelin can utilize these properties to prevent the progression of atherosclerosis also needs to be further clarified.

## 5. Conclusions

This study revealed that embelin is a PKC inhibitor with potent antiplatelet and antithrombotic effects. Embelin could effectively prevent PKC activation and subsequent activation of Akt and MAPKs in vitro and block thrombus formation in vivo. However, the further analysis is necessary to more accurately determine clinical therapeutic potential of embelin in all clinical thromboembolic events with disturbance of thrombocyte function.

## Supplementary Materials

The following are available online at https://www.mdpi.com/2077-0383/8/10/1724/s1.

## References

[1] van der Meijden P.E.J., Heemskerk J.W.M. (2019). Platelet biology and functions: New concepts and clinical perspectives. Nat. Rev. Cardiol..

[2] Versteeg H.H., Heemskerk J.W., Levi M., Reitsma P.H. (2013). New fundamentals in hemostasis. Physiol. Rev..

[3] Qin X., Meghana K., Sowjanya N.L., Sushma K.R., Krishna C.G., Manasa J., Sita G.J.A., Gowthami M., Honeyshmitha D., Srikanth G. (2019). Embelin attenuates cisplatin-induced nephrotoxicity: Involving inhibition of oxidative stress and inflammation in addition with activation of Nrf-2/Ho-1 pathway. Biofactors.

[4] Zhao Z.G., Tang Z.Z., Zhang W.K., Li J.G. (2015). Protective effects of embelin on myocardial ischemia-reperfusion injury following cardiac arrest in a rabbit model. Inflammation.

[5] Thippeswamy B.S., Nagakannan P., Shivasharan B.D., Mahendran S., Veerapur V.P., Badami S. (2011). Protective effect of embelin from Embelia ribes Burm. against transient global ischemia-induced brain damage in rats. Neurotox. Res..

[6] Xue Z., Ge Z., Zhang K., Sun R., Yang J., Han R., Peng M., Li Y., Li W., Zhang D. (2014). Embelin suppresses dendritic cell functions and limits autoimmune encephalomyelitis through the TGF-beta/beta-catenin and STAT3 signaling pathways. Mol. Neurobiol..

[7] Gupta R., Sharma A.K., Sharma M.C., Gupta R.S. (2012). Antioxidant activity and protection of pancreatic beta-cells by embelin in streptozotocin-induced diabetes. J. Diabetes.

[8] Naik S.R., Niture N.T., Ansari A.A., Shah P.D. (2013). Anti-diabetic activity of embelin: Involvement of cellular inflammatory mediators, oxidative stress and other biomarkers. Phytomedicine.

[9] Dai Y., Jiao H., Teng G., Wang W., Zhang R., Wang Y., Hebbard L., George J., Qiao L. (2014). Embelin reduces colitis-associated tumorigenesis through limiting IL-6/STAT3 signaling. Mol. Cancer Ther..

[10] Heo J.Y., Kim H.J., Kim S.M., Park K.R., Park S.Y., Kim S.W., Nam D., Jang H.J., Lee S.G., Ahn K.S. (2011). Embelin suppresses STAT3 signaling, proliferation, and survival of multiple myeloma via the protein tyrosine phosphatase PTEN. Cancer Lett..

[11] Peng M., Huang B., Zhang Q., Fu S., Wang D., Cheng X., Wu X., Xue Z., Zhang L., Zhang D. (2014). Embelin inhibits pancreatic cancer progression by directly inducing cancer cell apoptosis and indirectly restricting IL-6 associated inflammatory and immune suppressive cells. Cancer Lett..

[12] Dhanjal J.K., Nigam N., Sharma S., Chaudhary A., Kaul S.C., Grover A., Wadhwa R. (2014). Embelin inhibits TNF-alpha converting enzyme and cancer cell metastasis: Molecular dynamics and experimental evidence. BMC Cancer.

[13] Lee H., Ko J.H., Baek S.H., Nam D., Lee S.G., Lee J., Yang W.M., Um J.Y., Kim S.H., Shim B.S. (2016). Embelin Inhibits Invasion and Migration of MDA-MB-231 Breast Cancer Cells by Suppression of CXC Chemokine Receptor 4, Matrix Metalloproteinases-9/2, and Epithelial-Mesenchymal Transition. Phytother. Res..

[14] Lin K.H., Hsiao G., Shih C.M., Chou D.S., Sheu J.R. (2009). Mechanisms of resveratrol-induced platelet apoptosis. Cardiovasc. Res..

[15] Cattaneo M., Cerletti C., Harrison P., Hayward C.P.M., Kenny D., Nugent D., Nurden P., Rao A.K., Schmaier A.H., Watson S.P. (2013). Recommendations for the standardization of light transmission aggregometry: A consensus of the working party from the platelet physiology subcommittee of SSC/ISTH. J. Thromb. Haemost..

[16] Hechler B., Dupuis A., Mangin P.H., Gachet C. (2019). Platelet preparation for function testing in the laboratory and clinic: Historical and practical aspects. Res. Pract. Thromb. Haemost..

[17] Cazenave J.P., Ohlmann P., Cassel D., Eckly A., Hechler B., Gachet C. (2004). Preparation of washed platelet suspensions from human and rodent blood. Methods Mol. Biol..

[18] Lien L.M., Lin K.H., Huang L.T., Tseng M.F., Chiu H.C., Chen R.J., Lu W.J. (2017). Licochalcone A Prevents Platelet Activation and Thrombus Formation through the Inhibition of PLCgamma2-PKC, Akt, and MAPK Pathways. Int. J. Mol. Sci..

[19] Lin K.H., Kuo J.R., Lu W.J., Chung C.L., Chou D.S., Huang S.Y., Lee H.C., Sheu J.R. (2013). Hinokitiol inhibits platelet activation ex vivo and thrombus formation in vivo. Biochem. Pharmacol..

[20] Reagan-Shaw S., Nihal M., Ahmad N. (2008). Dose translation from animal to human studies revisited. FASEB J..

[21] Yacoub D., Theoret J.F., Villeneuve L., Abou-Saleh H., Mourad W., Allen B.G., Merhi Y. (2006). Essential role of protein kinase C delta in platelet signaling, alpha IIb beta 3 activation, and thromboxane A2 release. J. Biol. Chem..

[22] Cohen S., Braiman A., Shubinsky G., Isakov N. (2011). Protein kinase C-theta in platelet activation. FEBS Lett..

[23] Nikolovska-Coleska Z., Xu L., Hu Z., Tomita Y., Li P., Roller P.P., Wang R., Fang X., Guo R., Zhang M. (2004). Discovery of embelin as a cell-permeable, small-molecular weight inhibitor of XIAP through structure-based computational screening of a traditional herbal medicine three-dimensional structure database. J. Med. Chem..

[24] Konopatskaya O., Matthews S.A., Harper M.T., Gilio K., Cosemans J.M., Williams C.M., Navarro M.N., Carter D.A., Heemskerk J.W., Leitges M. (2011). Protein kinase C mediates platelet secretion and thrombus formation through protein kinase D2. Blood.

[25] O’Brien K.A., Stojanovic-Terpo A., Hay N., Du X. (2011). An important role for Akt3 in platelet activation and thrombosis. Blood.

[26] Woulfe D.S. (2010). Akt signaling in platelets and thrombosis. Expert Rev. Hematol..

[27] Kim S., Jin J., Kunapuli S.P. (2004). Akt activation in platelets depends on Gi signaling pathways. J. Biol. Chem..

[28] Adam F., Kauskot A., Nurden P., Sulpice E., Hoylaerts M.F., Davis R.J., Rosa J.P., Bryckaert M. (2010). Platelet JNK1 is involved in secretion and thrombus formation. Blood.

[29] Lopez-Bergami P., Habelhah H., Bhoumik A., Zhang W., Wang L.H., Ronai Z. (2005). RACK1 mediates activation of JNK by protein kinase C [corrected]. Mol. Cell.

[30] Mauro A., Ciccarelli C., De Cesaris P., Scoglio A., Bouche M., Molinaro M., Aquino A., Zani B.M. (2002). PKCalpha-mediated ERK, JNK and p38 activation regulates the myogenic program in human rhabdomyosarcoma cells. J. Cell Sci..

[31] Ahn K.S., Sethi G., Aggarwal B.B. (2007). Embelin, an inhibitor of X chromosome-linked inhibitor-of-apoptosis protein, blocks nuclear factor-kappaB (NF-kappaB) signaling pathway leading to suppression of NF-kappaB-regulated antiapoptotic and metastatic gene products. Mol. Pharmacol..

[32] Zhou Z., Gushiken F.C., Bolgiano D., Salsbery B.J., Aghakasiri N., Jing N., Wu X., Vijayan K.V., Rumbaut R.E., Adachi R. (2013). Signal transducer and activator of transcription 3 (STAT3) regulates collagen-induced platelet aggregation independently of its transcription factor activity. Circulation.

[33] Malaver E., Romaniuk M.A., D’Atri L.P., Pozner R.G., Negrotto S., Benzadon R., Schattner M. (2009). NF-kappaB inhibitors impair platelet activation responses. J. Thromb. Haemost..

[34] Lu W.J., Lin K.C., Huang S.Y., Thomas P.A., Wu Y.H., Wu H.C., Lin K.H., Sheu J.R. (2014). Role of a Janus kinase 2-dependent signaling pathway in platelet activation. Thromb. Res..

[35] Lu W.J., Lin K.H., Hsu M.J., Chou D.S., Hsiao G., Sheu J.R. (2012). Suppression of NF-kappaB signaling by andrographolide with a novel mechanism in human platelets: Regulatory roles of the p38 MAPK-hydroxyl radical-ERK2 cascade. Biochem. Pharmacol..

[36] Oleksowicz L., Mrowiec Z., Zuckerman D., Isaacs R., Dutcher J., Puszkin E. (1994). Platelet activation induced by interleukin-6: Evidence for a mechanism involving arachidonic acid metabolism. Thromb. Haemost..

[37] Joshi R., Kamat J.P., Mukherjee T. (2007). Free radical scavenging reactions and antioxidant activity of embelin: Biochemical and pulse radiolytic studies. Chem. Biol. Interact..

[38] Singh D., Singh R., Singh P., Gupta R.S. (2009). Effects of embelin on lipid peroxidation and free radical scavenging activity against liver damage in rats. Basic Clin. Pharmacol. Toxicol..

[39] Begonja A.J., Gambaryan S., Geiger J., Aktas B., Pozgajova M., Nieswandt B., Walter U. (2005). Platelet NAD(P)H-oxidase-generated ROS production regulates alphaIIbbeta3-integrin activation independent of the NO/cGMP pathway. Blood.

[40] Jang J.Y., Min J.H., Chae Y.H., Baek J.Y., Wang S.B., Park S.J., Oh G.T., Lee S.H., Ho Y.S., Chang T.S. (2014). Reactive oxygen species play a critical role in collagen-induced platelet activation via SHP-2 oxidation. Antioxid. Redox Signal..

[41] Hansson G.K. (2005). Inflammation, atherosclerosis, and coronary artery disease. N. Engl. J. Med..

[42] Hansson G.K., Libby P. (2006). The immune response in atherosclerosis: A double-edged sword. Nat. Rev. Immunol..

[43] Libby P., Ridker P.M., Hansson G.K. (2009). Inflammation in atherosclerosis: From pathophysiology to practice. J. Am. Coll. Cardiol..

[44] Moore K.J., Tabas I. (2011). Macrophages in the pathogenesis of atherosclerosis. Cell.

[45] Hansson G.K., Robertson A.K., Soderberg-Naucler C. (2006). Inflammation and atherosclerosis. Annu. Rev. Pathol..

[46] Lindemann S., Kramer B., Seizer P., Gawaz M. (2007). Platelets, inflammation and atherosclerosis. J. Thromb. Haemost..

[47] Zimman A., Podrez E.A. (2010). Regulation of platelet function by class B scavenger receptors in hyperlipidemia. Arterioscler. Thromb. Vasc. Biol..

[48] Huo Y., Schober A., Forlow S.B., Smith D.F., Hyman M.C., Jung S., Littman D.R., Weber C., Ley K. (2003). Circulating activated platelets exacerbate atherosclerosis in mice deficient in apolipoprotein E. Nat. Med..

[49] Massberg S., Brand K., Gruner S., Page S., Muller E., Muller I., Bergmeier W., Richter T., Lorenz M., Konrad I. (2002). A critical role of platelet adhesion in the initiation of atherosclerotic lesion formation. J. Exp. Med..

[50] Pitsilos S., Hunt J., Mohler E.R., Prabhakar A.M., Poncz M., Dawicki J., Khalapyan T.Z., Wolfe M.L., Fairman R., Mitchell M. (2003). Platelet factor 4 localization in carotid atherosclerotic plaques: Correlation with clinical parameters. Thromb. Haemost..

[51] Henn V., Slupsky J.R., Grafe M., Anagnostopoulos I., Forster R., Muller-Berghaus G., Kroczek R.A. (1998). CD40 ligand on activated platelets triggers an inflammatory reaction of endothelial cells. Nature.

[52] Mach F., Schonbeck U., Sukhova G.K., Atkinson E., Libby P. (1998). Reduction of atherosclerosis in mice by inhibition of CD40 signalling. Nature.

[53] Lutgens E., Gorelik L., Daemen M.J., de Muinck E.D., Grewal I.S., Koteliansky V.E., Flavell R.A. (1999). Requirement for CD154 in the progression of atherosclerosis. Nat. Med..

[54] Chaudhari H.S., Bhandari U., Khanna G. (2012). Preventive effect of embelin from embelia ribes on lipid metabolism and oxidative stress in high-fat diet-induced obesity in rats. Planta Med..

[55] Fan H.C., Fernandez-Hernando C., Lai J.H. (2014). Protein kinase C isoforms in atherosclerosis: Pro- or anti-inflammatory?. Biochem. Pharmacol..

[56] Kong L., Shen X., Lin L., Leitges M., Rosario R., Zou Y.S., Yan S.F. (2013). PKCbeta promotes vascular inflammation and acceleration of atherosclerosis in diabetic ApoE null mice. Arterioscler. Thromb. Vasc. Biol..

[57] Durpes M.C., Morin C., Paquin-Veillet J., Beland R., Pare M., Guimond M.O., Rekhter M., King G.L., Geraldes P. (2015). PKC-beta activation inhibits IL-18-binding protein causing endothelial dysfunction and diabetic atherosclerosis. Cardiovasc. Res..

[58] de Winther M.P., Kanters E., Kraal G., Hofker M.H. (2005). Nuclear factor kappaB signaling in atherogenesis. Arterioscler. Thromb. Vasc. Biol..

[59] Pamukcu B., Lip G.Y., Shantsila E. (2011). The nuclear factor—Kappa B pathway in atherosclerosis: A potential therapeutic target for atherothrombotic vascular disease. Thromb. Res..

[60] Schuett H., Luchtefeld M., Grothusen C., Grote K., Schieffer B. (2009). How much is too much? Interleukin-6 and its signalling in atherosclerosis. Thromb. Haemost..

[61] Dutzmann J., Daniel J.M., Bauersachs J., Hilfiker-Kleiner D., Sedding D.G. (2015). Emerging translational approaches to target STAT3 signalling and its impact on vascular disease. Cardiovasc. Res..

[62] Chen Q., Lv J., Yang W., Xu B., Wang Z., Yu Z., Wu J., Yang Y., Han Y. (2019). Targeted inhibition of STAT3 as a potential treatment strategy for atherosclerosis. Theranostics.

