# Supplementary material for: Embelin as a Novel Inhibitor of PKC in the Prevention of Platelet Activation and Thrombus Formation
Source: J Clin Med. 2019 Oct 18;8(10):1724. doi: 10.3390/jcm8101724 (PMC6832570; doi:10.3390/jcm8101724)
Supplement: Supplementary file 1 [file jcm-08-01724-s001.pdf]

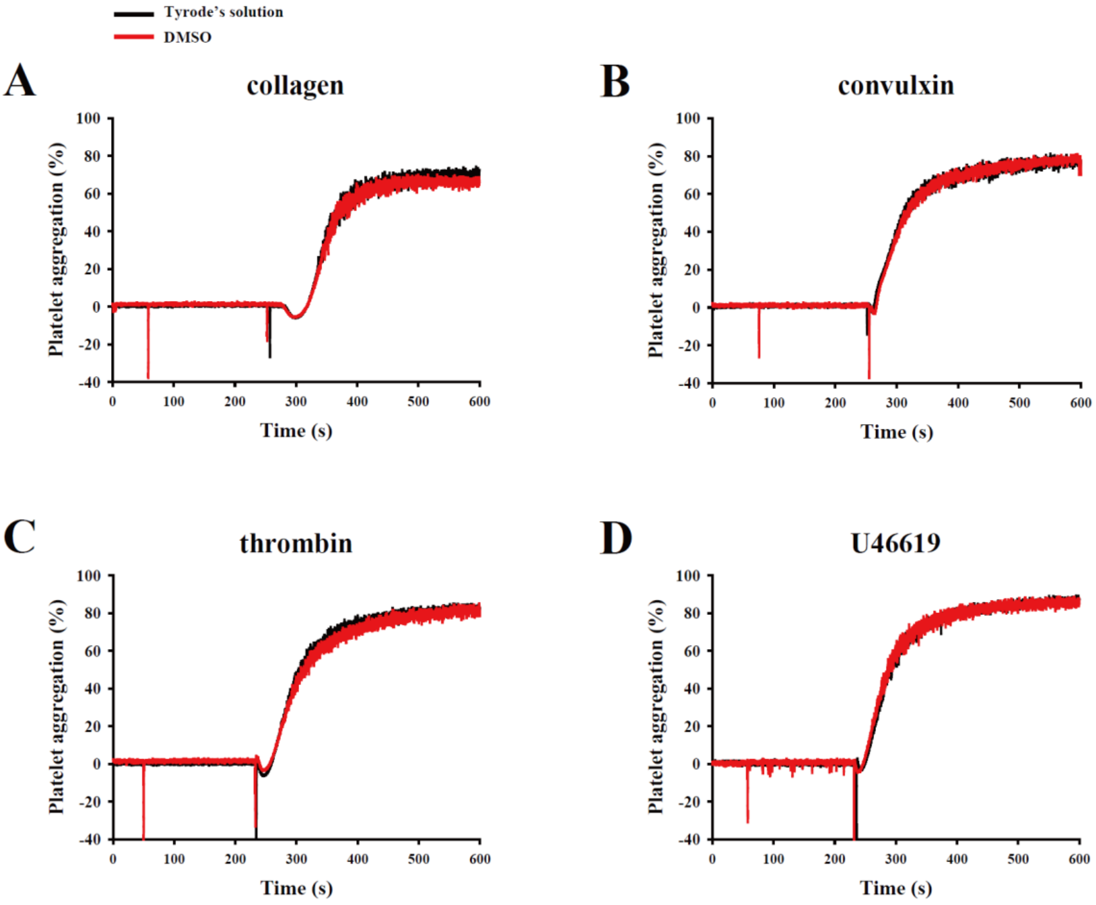

Supplemental Figure 1. Human washed platelets (3.6 × 10^8 cells/mL) were pretreated with Tyrode's solution (control) or dimethyl sulfoxide (DMSO) (solvent control), followed by stimulation with (**A**) collagen (1 μg/mL), (**B**) convulxin (10 ng/mL), (**C**) thrombin (0.02 U/mL), and (**D**) U46619 (1 μM) to induce platelet aggregation.

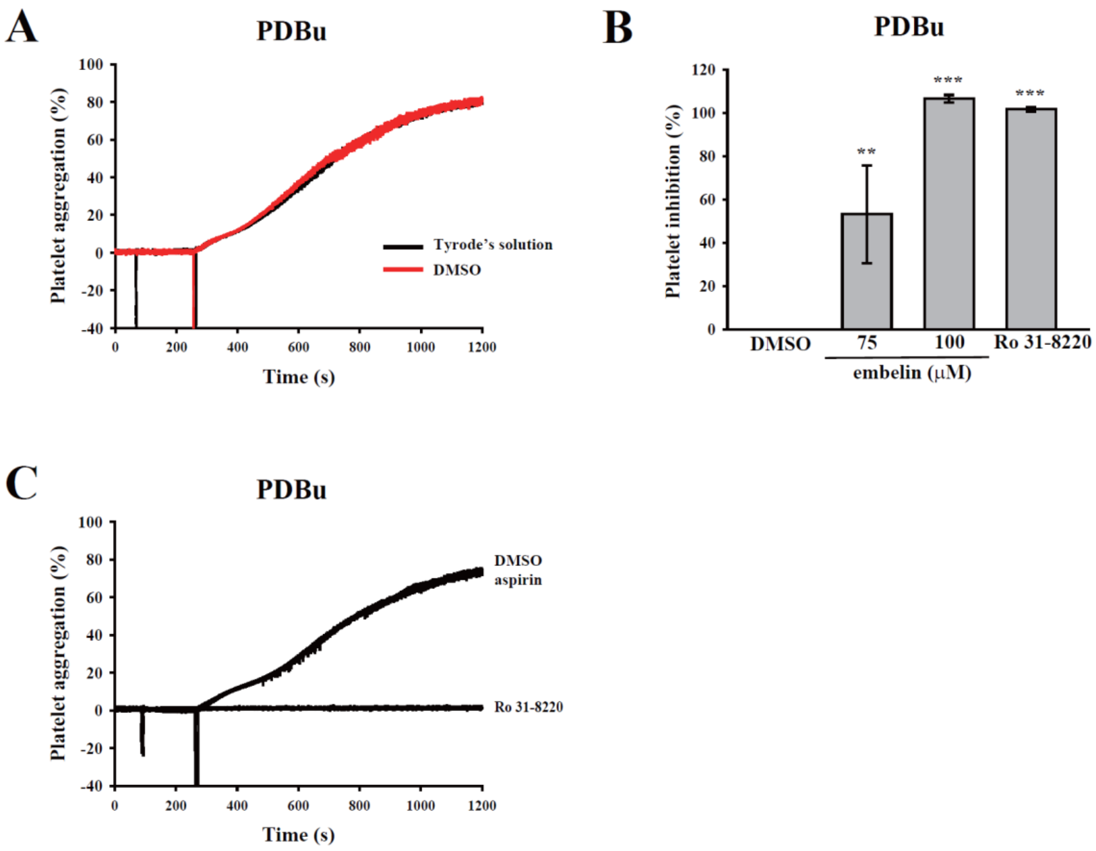

Supplemental Figure 2. Human washed platelets (3.6 × 10^8 cells/mL) were pretreated with Tyrode's solution (control), DMSO (solvent control), embelin (75 and 100 μM), the PKC inhibitor Ro 31-8220 (2 μM), or aspirin (100 μM), followed by the stimulation of phorbol 12,13-dibutyrate (PDBu) (150 nM) to induce platelet aggregation. Data (**B**) are presented as means ± standard error of the mean (SEM) (n = 3). **$P < 0.01$ and ***$P < 0.001$, compared with the DMSO (solvent control) group.
